# Genome wide association study reveals new genes for resistance to striped stem borer in rice (*Oryza sativa* L.)

**DOI:** 10.3389/fpls.2024.1466857

**Published:** 2024-09-13

**Authors:** Xing Xiang, Shuhua Liu, Yuewen He, Deqiang Li, Andrews Danso Ofori, Abdul Ghani Kandhro, Tengda Zheng, Xiaoqun Yi, Ping Li, Fu Huang, Aiping Zheng

**Affiliations:** ^1^ State Key Laboratory of Crop Gene Exploration and Utilization in Southwest China, Sichuan Agricultural University, Chengdu, China; ^2^ College of Agronomy, Sichuan Agricultural University, Chengdu, China; ^3^ Guangan Vocational & Technical College, Guangan, China

**Keywords:** rice, striped stem borer, GWAS, resistance genes, SNP

## Abstract

Rice is one of the most important food crops in the world and is important for global food security. However, damage caused by striped stem borer (SSB) seriously threatens rice production and can cause significant yield losses. The development and use of resistant rice varieties or genes is currently the most effective strategy for controlling SSB. We genotyped 201 rice samples using 2849855 high-confidence single nucleotide polymorphisms (SNPs). We conducted a genome-wide association study (GWAS) based on observed variation data of 201 rice cultivars resistant to SSB. We obtained a quantitative trait locus (QTL)-*qRSSB4* that confers resistance to SSB. Through annotation and analysis of genes within the *qRSSB4* locus, as well as qRT-PCR detection in resistant rice cultivars, we ultimately selected the candidate gene *LOC_Os04g34140* (named *OsRSSB4*) for further analysis. Next, we overexpressed the candidate gene *OsRSSB4* in Nipponbare through transgenic methods, resulting in *OsRSSB4* overexpressing lines (*OsRSSB4OE*). In addition, we evaluated the insect resistance of *OsRSSB4OE* lines using wild type (Nipponbare) as a control. The bioassay experiment results of live plants showed that after 20 days of inoculation with SSB, the withering heart rate of *OsRSSB4OE-34* and *OsRSSB4OE-39* lines was only 8.3% and 0%, with resistance levels of 1 and 0, respectively; however, the withering heart rate of the wild-type reached 100%, with a resistance level of 9. The results of the *in vitro* stem bioassay showed that, compared with the wild-type, the average corrected mortality rate of the SSB fed on the *OsRSSB4OE* line reached 94.3%, and the resistance reached a high level. In summary, we preliminarily confirmed that *OsRSSB4* positively regulates the defense of rice against SSB. This research findings reveal new SSB resistance gene resources, providing an important genetic basis for SSB resistance breeding in rice crops.

## 1 Introduction

As the world’s largest food crop, rice (Oryza sativa L.) is a staple food for nearly 3.5 billion people worldwide, accounting for approximately half of the world’s population (Zhang et al., 2020). According to rough estimates, rice production must increase at a rate of 1% per year to provide sufficient rice for the growing populations of rice-consuming countries. However, global rice production is seriously threatened by Lepidopteran pests, resulting in severe yield loss (Xia et al., 2010; Li et al., 2016). Among them, the striped stem borer (SSB) poses a particularly serious threat to rice, causing an annual economic loss of approximately 1.8 billion dollars (Sheng et al., 2003). Generally speaking, striped stem borers only occur twice a year, but with global warming causing higher temperatures in winter and spring, the severity of their harm is increasing (Xiang et al., 2023). At present, stem borer control relies heavily on chemical insecticides (Lou et al., 2013). However, long-term overuse of chemical insecticides has led to a reduction in the number of beneficial organisms in agricultural ecosystems, environmental pollution, and food safety issues becoming increasingly common (Chagnon et al., 2015). Therefore, cultivating and applying insect-resistant rice varieties is the most effective way to control this pest and to ensure food security.

To date, only over ten resistance genes related to SSB have been reported in rice plants. Among them, the positive regulatory genes included *OsHI-XIP* (Xin et al., 2014), *OsACS2* (Lu et al., 2014), *OsWRKY70* (Li et al., 2015), *OsMPK4* (Liu et al., 2018), *OsAOC*, *OsOPR3* (Guo et al., 2014), *OsAPIP4* (Liu et al., 2021), *OsLRR-RLK1* (Hu et al., 2018), *OsMPK3* (Wang et al., 2013), *OsHI-LOX* (Zhou et al., 2009), *OsERF3* (Lu et al., 2011b), *OsAOS1*, and *OsAOS2* (Zeng et al., 2021); Negative regulatory genes included *OsNPR1* (Li et al., 2013), *OsCYP71A1* (Lu et al., 2018), *OM64* (Guo et al., 2020), *Osr9-LOX1* (Zhou et al., 2014), *OsHPL3* (Tong et al., 2012), and *OsWRKY53* (Hu et al., 2015). Rice borer resistance is a quantitative trait, similar to most important agronomic traits, such as yield, quality, and drought resistance, which are controlled by minor effect polygenes, namely quantitative trait genes or quantitative trait loci (QTLs), and manifest as quantitative inheritance. In genetically isolated or variable populations, QTLs are statistically significant associations between allele variations at specific loci and phenotypic traits that exhibit continuous variation (St Clair, 2010). However, the key QTLs for SSB resistance in rice plants have not yet been reported. However, the detection and identification of insect-resistant breeding is relatively difficult, making the breeding of SSB-resistant rice varieties slow. In recent years, genetically modified technology has been used to introduce exogenous genes into rice in order to obtain insect-resistant varieties more quickly, such as Huahui 1 (transgenic with Cry1Ab/1Ac fusion gene), but its biological safety issues have been questioned (Wang et al., 2016). Therefore, detecting new endogenous resistance genes and QTLs in many rice cultivars is crucial for the successful cultivation of rice that can resist SSB.

With the advancement of sequencing technology, genome-wide association studies (GWAS) based on high-density single nucleotide polymorphisms (SNPs) have become a valuable method for identifying the genetic basis of phenotypic variation (Lipka et al., 2012; Burghardt et al., 2017). To study the genetic diversity of drought resistance in rice, Sun et al. (2022). evaluated the drought stress phenotype of 271 rice germplasms under field drought conditions and identified seven SNPs significantly associated with drought through GWAS. Researchers inoculated 584 rice materials with three varieties of rice blast fungus and identified 27 loci associated with rice blast resistance using genome-wide association studies (Liu et al., 2020). Wang et al. (2021). conducted a genome-wide association study on sheath blight resistance in 259 different rice varieties. Based on the best linear unbiased prediction (BLUP) value, 1396 SNP loci were found to be significantly correlated with sheath blight resistance (log_10_ *P* ≥ 6). Therefore, it is feasible to conduct genome-wide association studies using large amounts of insect-resistant phenotype data from rice populations.

In our study, 201 rice varieties were artificially introduced into striped stem borer larvae during the peak tillering stage to evaluate their SSB resistance. Based on previous research (Wang et al., 2021) by our group, we conducted GWAS for SSB resistance using 2849855 high-confidence SNPs (missing data < 20%; minor allele frequency [MAF] > 1%). Moreover, we overexpressed candidate genes in the rice variety Nipponbare using genetic transformation methods to verify their gene functions. Our results enabled the detection of candidate genes related to SSB resistance in these rice cultivars. This provides important genetic resources for breeding rice with insect resistance.

## 2 Materials and methods

### 2.1 Plant materials and tested insects

In this study, 201 rice cultivars were used. These varieties originate from multiple geographical locations, including China, Senegal, Mexico, Malaysia, Colombia, and Brazil. Among them, 108 varieties were preserved by the International Rice Research Institute and 93 varieties were preserved by the College of Agronomy of Sichuan Agricultural University. Detailed information for each variety is provided in **Supplementary Table S1**. To evaluate the SSB resistance of the rice varieties, seeds of all 201 rice varieties were sown in experimental fields (Wenjiang Huihe Base of Sichuan Agricultural University). After 30 days of age, transplanting was carried out with one row of 10 plants per variety (row spacing of 30 cm and plant spacing of 20 cm).

The striped stem borer was collected from the rice field of Huihe Base, Sichuan Agricultural University, in 2019. In an artificial climate chamber (temperature 27 ± 1°C, relative humidity 70-80%, lighting time 16L: 8D), the artificial feeding method described by Kamano (1973) was used to cultivate the striped stem borer. Every three generations, the stems of rice variety TN1 were used to feed the borers for one generation (with the aim of maintaining a high vitality state in the striped stem borer population), and this process was continued for over 30 generations. First-instar larvae (ant borers) were used in this experiment.

### 2.2 Field inoculation and determination of insect resistance

According to the standards of the International Rice Institute (with slight modifications) (Pathak et al., 1971), we identified the insect resistance of the rice varieties in the field. First, during the peak tillering period of rice, we selected three healthy and well-grown rice plants from each variety. Subsequently, we removed the weak tillers from each rice plant and counted the remaining number of tillers. Third, we used a small soft bristled brush to inoculate the corresponding number of ant borers (half of the tiller number of rice plants) onto each rice variety, with three replicate sets for each rice variety. Rice variety TN1 was used as the blank control (susceptible control). Finally, to ensure the accuracy of the experiment, natural enemies, pests, and insect eggs were removed prior to inoculation. Thirty days later, we counted the number of withered seedlings and booths for 201 rice varieties. The damage index is the ratio of the total number of withered seedlings and withered booths to the number of tillers in the rice plant, and the corrected damage index (D) is the ratio of the damage index of the rice plant to the damage index of the blank control TN1. The average damage index of each variety was calculated based on three rice plant lines. The data were processed using Microsoft Excel 2021. Statistical analysis was conducted on the corrected damage index between different rice subpopulations using analysis of variance (ANOVA), followed by the Scheffe multiple comparison test in SPSS v26.0 (IBM Corp., USA). According to Heinrichs’ (Heinrichs et al., 1985) rating criteria for rice insect resistance (D=0, represented by 0, representing high resistance; 0<D≤ 20%, represented by 1, representing resistance; 20%<D ≤ 40%, represented by 3, representing moderate resistance; 40%<D ≤ 60%, represented by 5, representing insect tolerance; 60%<D ≤ 80%, represented by 7, representing sensitivity; D>80%, represented by 9, representing high sensitivity), record the insect resistance scores of each rice cultivar.

For the insect resistance evaluation of transgenic rice plants, we conducted bioassay experiments on both live plants and detached stems. For the bioassay of live plants, we selected two transgenic rice plants and one wild-type rice plant (Nipponbare) that were at the mid-tillering stage and had similar growth vigor; Then, we removed the smaller tillers from the rice plants and counted the remaining tillers; Finally, we inoculated the rice plants with the ant borers, with the number of larvae corresponding to half of the tiller count of each rice plant; After 20 days, we counted the withered heart rate of each rice plant. For the bioassay of detached stems, we selected four control plants (Nipponbare) and four transgenic rice plants to be tested, and then cut 5 cm long stems from the same position on each plant for later use; Second, we gently attached 10 ant borers to each detached stem using a soft bristled brush, and placed the infected stem into a 20×150 mm test tube with 2 mm deep water at the bottom, sealed with filter paper; The test tubes were then placed at room temperature; Thirdly, on the 7th day after insect inoculation, the number of dead larvae in the stem was dissected and recorded separately. Subsequently, the larval mortality rate (i.e., the ratio of dead larvae to the total number of infected larvae) and corrected larval mortality rate (i.e., the ratio of the larval mortality rate of each rice variety minus the mortality rate of the control variety to 1 minus the mortality rate of the control variety) were calculated. Using the mortality rate (d) of SSB larvae as an indicator, the evaluation criteria for rice insect resistance are as follows: d=100%, represented by 0, indicating high resistance; d>80%, represented by 1, indicating resistance; 60%<d ≤ 80%, represented by 3, indicating moderate resistance; 40%<d ≤ 60%, represented by 5, indicating insect tolerance; 20%<d ≤ 40%, represented by 3, indicating susceptibility to insects; 0<d ≤ 20%, represented by 1, indicating high susceptibility.

### 2.3 DNA extraction and sequencing

Young leaves of 21-day-old seedlings of each variety were sampled for genomic DNA extraction. Total genomic DNA was prepared using the cetyltrimethylammonium bromide (CTAB) method (Uzunova et al., 1995). Genomic DNA samples from all 201 rice lines were fragmented by sonication to a size of 350bp, and DNA fragments were then end-polished, A-tailed, and ligated with full-length adapters for Illumina sequencing with further PCR amplification. Reads containing adapter sequence stretches of -Ns and low-quality scores were excluded from the raw data. The remaining high-quality paired-end reads were mapped to the Nipponbare reference genome using Burrows-Wheeler Aligner software with the command ‘mem -t 4 -k 32 –M’ (Li and Durbin, 2010). After alignment, genomic variants (in GVCF format for each accession) were identified with the GVCF model using Genome Analysis Toolkit (GATK) software (McKenna et al., 2010).

### 2.4 GWAS analysis

Only SNPs with sequencing depth ≥ 4, missing rate < 0.2, and MAF ≥ 0.01 were used in the GWAS. GEMMA(http://www.xzlab.org/software.html) software is used to analyze SNP-GWAS data (Zhou and Stephens, 2012; Zhou et al., 2013; Zhou and Stephens, 2014; Zhou, 2017). In the GWAS analysis, individual kinship and population stratification were the main factors causing false-positive associations. Therefore, a Mixed Linear Model (MLM) was used for trait association analysis, with population genetic structure as a fixed effect and individual kinship as a random effect, to correct for the influence of population structure and individual kinship: y=Xα+Zβ+Wμ+e [y is a phenotypic trait, X is a fixed effects index matrix, α is an estimating parameter for fixed effects, Z is the index matrix of SNP, β is the effect of SNP, the matrix where W represents random effects, μ is the predicted random individuals, and e is a random residual that follows e~(0, δe2)]. In addition, a significant *P*-value threshold (*P*<10^-6^) was set to control the whole-genome type 1 error rate, which was calculated by rounding to 1/n (total SNPs) ≈ 6 to screen for potential candidate SNPs.

### 2.5 Transgenic analysis

In order to further identify the function of candidate genes for SSB resistance, an OE line was generated under the background of Nipponbare (susceptible cultivar). To generate the OE line, gene-specific primers were used to amplify the coding sequence (CDS) of each candidate gene from Nipponbare using PCR. The cDNA product was then cloned downstream of the CaMV 35S promoter in pBWA (V) HS, and the resulting vector was introduced into *Agrobacterium tumefaciens* strain GV3101. The transformation of rice was completed by Wuhan Boyuan Biotechnology Co., Ltd., China. To identify the OE line, the expression levels of candidate genes in transgenic rice were analyzed using qRT-PCR. T4 generation transgenic rice was used for the inoculation experiments. The primers used in this study are listed in **Supplementary Table S5**.

### 2.6 qRT−PCR

The relative expression levels of candidate genes in rice were studied using qRT-PCR. Select one rice plant (Aituogu151) at the peak tillering stage with healthy growth, and inoculate each tiller with one second-instar larva of striped stem borer. After 12 hours, cut 2 cm (near the borer holes) of the stem from three tillers with borer holes, immediately cool them in liquid nitrogen, and store them at -80°C. The extraction and reverse transcription of total RNA were performed using a reagent kit from Beijing TransGen Biotech Co., Ltd. The PCR reaction volume was 10 µL, with 1 µL of cDNA template and 0.25 µL of forward and reverse gene-specific primers. Each PCR was performed in four replicates. The β-Actin gene was used as an internal control for data standardization. The 2^−ΔΔCt^ method was used to calculate gene expression levels. **Supplementary Table S5** lists the primers used in this experiment.

## 3 Results

### 3.1 Genome variation

**Figure 1:**
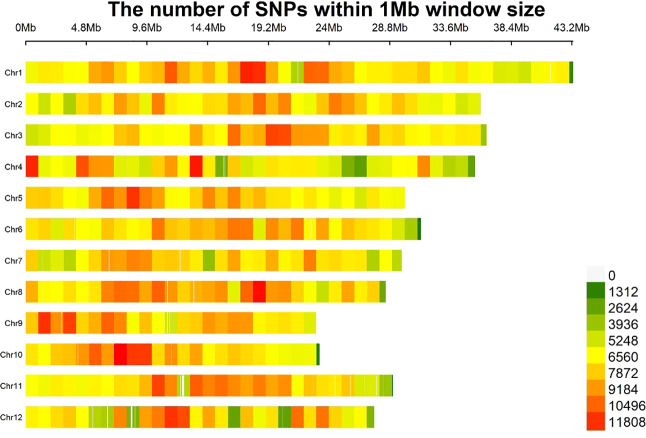
Distribution density of SNP loci on various chromosomes.

To identify SSB resistance genes in rice, we sequenced 201 rice varieties (**Supplementary Table S1**) using an Illumina Hi-Seq platform. The raw readings are presented in **Supplementary Table S2**. After mapping to the Nipponbare genome, 2849855 high-confidence SNPs were obtained (missing data < 20%; minor allele frequency [MAF] > 1%) (**Table 1**; **Figure 1**). Among the 2849855 high-confidence SNPs, 1130951, 548458, 359036, 458738, and 314291 were located in the intergenic, exonic, upstream, intronic, and downstream regions, respectively (**Table 1**). In the coding sequence (CDS), 16207 stop gains, 222512 synonymous, 308670 non-synonymous, 2811 splices, and 1069 stop-loss SNPs were identified (**Table 1**). In addition, there were 2046787 SNPs of the Ts (transitions) type and 803068 SNPs of the Tv (transversions) type. The ratio of Ts to Tv is 2.549, indicating that the bias during the variant call process is within a reasonable range. The number of SNPs in the 12 rice chromosomes ranges from 189859 (Chr. 9) to 330608 (Chr. 1), with Chr. 8 (8.35 SNPs/kb), which had the highest SNP frequencies (**Table 2**; **Figure 1**). This SNP dataset contained 201 rice varieties, providing abundant resources for the molecular improvement of SSB resistance in rice.

**Table 1:** Results of filtered SNP annotation.

Category		Number of SNPs
Upstream		359036
Exonic	Stop gain	16207
	Stop loss	1069
	Synonymous	222512
	Non-synonymous	308670
Intronic		458738
Splicing		2811
Downstream		314291
upstream/downstream		35570
Intergenic		1130951
ts		2046787
tv		803068
ts/tv		2.549
Total		2849855

**Table 2:** Number of SNPs in 201 rice accessions using the sequenced data mapped to the Nipponbare reference genome.

Chromosome	length(bp)	SNP number (MAF>1%, integrity>0.8)	SNP number/per Kb
Chr1	43270923	330608	7.640419411
Chr2	35937250	266983	7.429143855
Chr3	36413819	276357	7.589344035
Chr4	35502694	240515	6.774556320
Chr5	29958434	233005	7.777609471
Chr6	31248787	239110	7.651817013
Chr7	29697621	217667	7.329442315
Chr8	28443022	237516	8.350589470
Chr9	23012720	189859	8.250176424
Chr10	23207287	191801	8.264688587
Chr11	29021106	225247	7.761489173
Chr12	27531856	201187	7.307425987

### 3.2 Phenotypic variation among rice varieties

**Figure 2:**
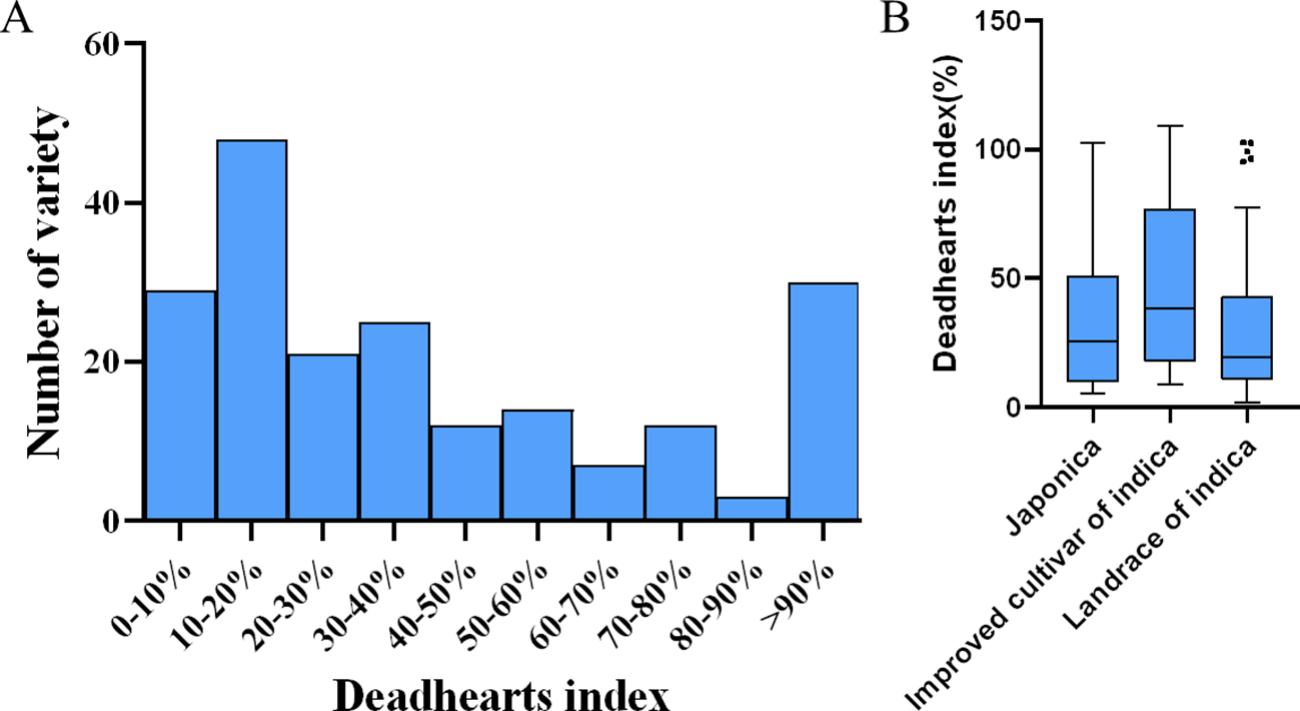
Sensory and resistance responses of 201 rice lines inoculated with SSB. **(A)** Frequency distribution of deadheart index after inoculation of 201 rice lines with SSB. **(B)** Box plot of resistance phenotype data of three rice subgroups to SSB.

All 201 rice varieties were inoculated with the corresponding number of 1st instar larvae of the SSB during the peak tillering period, and the resistance phenotype data of each cultivar were statistically analyzed (**Supplementary Table S1**). According to **Figure 2A**, the cultivars with a withered heart index (D) in the range of 0-10% have the highest number, reaching 71; The next two intervals are 10 < D ≤ 20% and D > 90%, which contain 48 and 30 rice cultivars, respectively; The average withered heart index is 34.72%; The wide range of D values observed in different rice cultivars indicates a significant correlation between genotype variation and rice resistance to SSB. From **Figure 2B**, it can be seen that the distribution of withered heart index data for landrace of indica is relatively concentrated, while the distribution of withered heart index data for improved cultivar of indica is relatively scattered (i.e. with large data fluctuations), indicating that artificial selection has not yet been successful in rice insect resistance breeding; In addition, the median withering index of japonica, landrace of indica, and improved cultivar of indica were 25.73%, 19.50%, and 38.13%, respectively, and the median withering index of the three types of rice varieties was closer to the lower quartile, belonging to a right skewed distribution.

### 3.3 GWAS for resistance to the SSB

**Figure 3:**
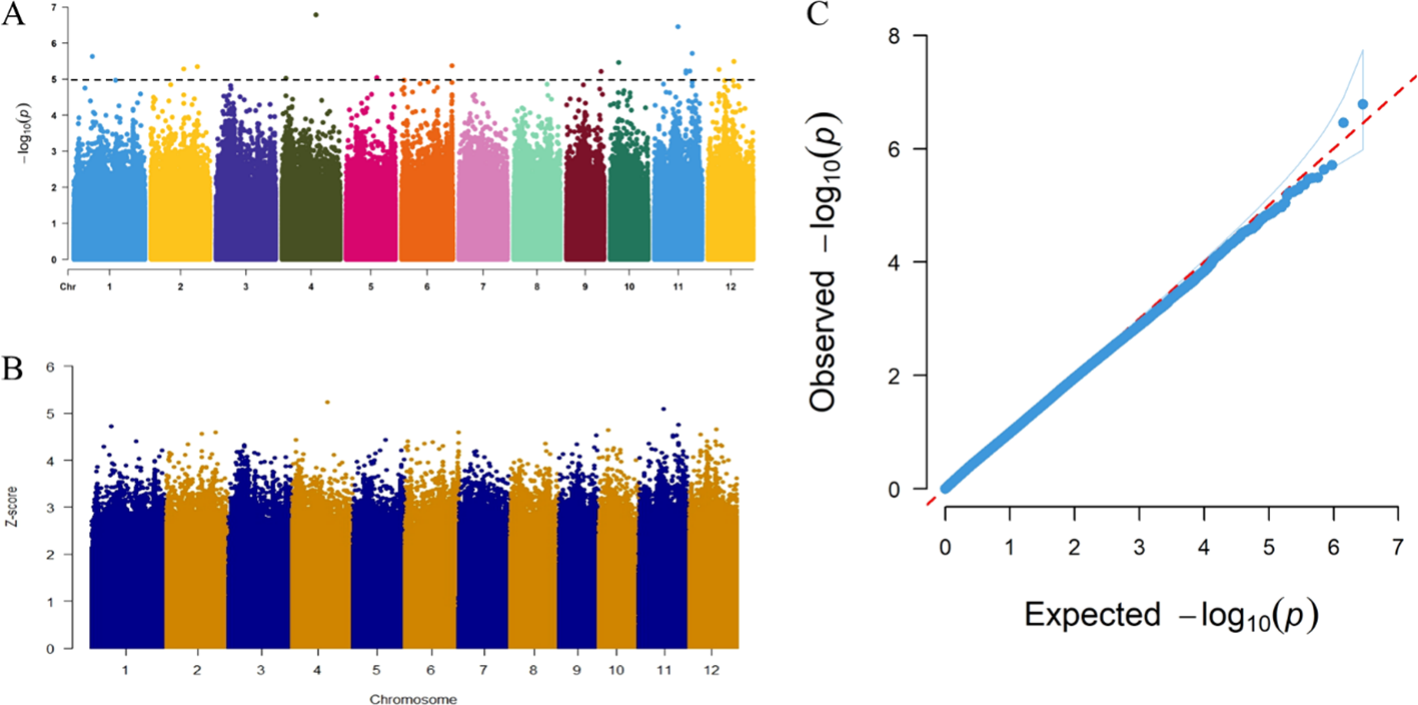
Manhattan plot and quantile-quantile (QQ) plot obtained from a genome-wide association study (GWAS) on rice resistance to SSB. **(A)** Manhattan plot of the resistance trait (withered heart index) of the striped stem borer (which is a genetic marker effect value, i.e., the whole-genome *P*-values sorted by physical location on the chromosome by F-test). **(B)** Manhattan plot after Z-score standardized *P*-values. **(C)** QQ plot of the rice resistance traits of the striped stem borer (showing the distribution of actual *P* values and expected *P* values based on the null hypothesis of correlation).

Based on 2849855 high-confidence SNPs, GWAS was performed using a mixed linear model (MLM) to analyze the phenotypic data (withered heart index) of resistance traits. As shown in **Figure 3C**, the predicted line in the Q-Q graph is a 45°dashed line originating from the origin, indicating that the resistance trait is not caused by population stratification. Through GWAS analysis, we obtained a total of 17 SNP loci (−log10*P* ≥ 5) that were significantly associated with SSB resistance, as well as their corresponding genotype variation data in 201 cultivars (**Figure 3A**; **Supplementary Table S4**). Interestingly, two pairs of SNP loci (ID 11 and 12; ID 16 and 17) belonged to the inclusion relationships (**Supplementary Table S4**). In addition, the strongest signal, ID5 (peak value=6.784146511), was observed on chromosome 4 (**Figure 3A**). Next, we standardized the *P*-values of all SNP sites using the Z-score, and then plotted the obtained Z-score values in a Manhattan plot (**Figure 3B**). As shown in **Figure 3B**, ID5 on chromosome 4 also had the highest Z-score. Therefore, we selected the lead SNP (ID5) on chromosome 4 for subsequent analysis. Based on the linkage disequilibrium decay rate of up to 194 kb in rice (Wang et al., 2021), we selected a region of approximately 194 kb upstream and downstream of the leading SNP position (20.752349 Mb) on chromosome 4 as candidate loci (referred to as *qRSSB4*) to narrow down the region containing causal genes.

### 3.4 Analysis of candidate genes within the *qRSSB4* locus

**Figure 4:**
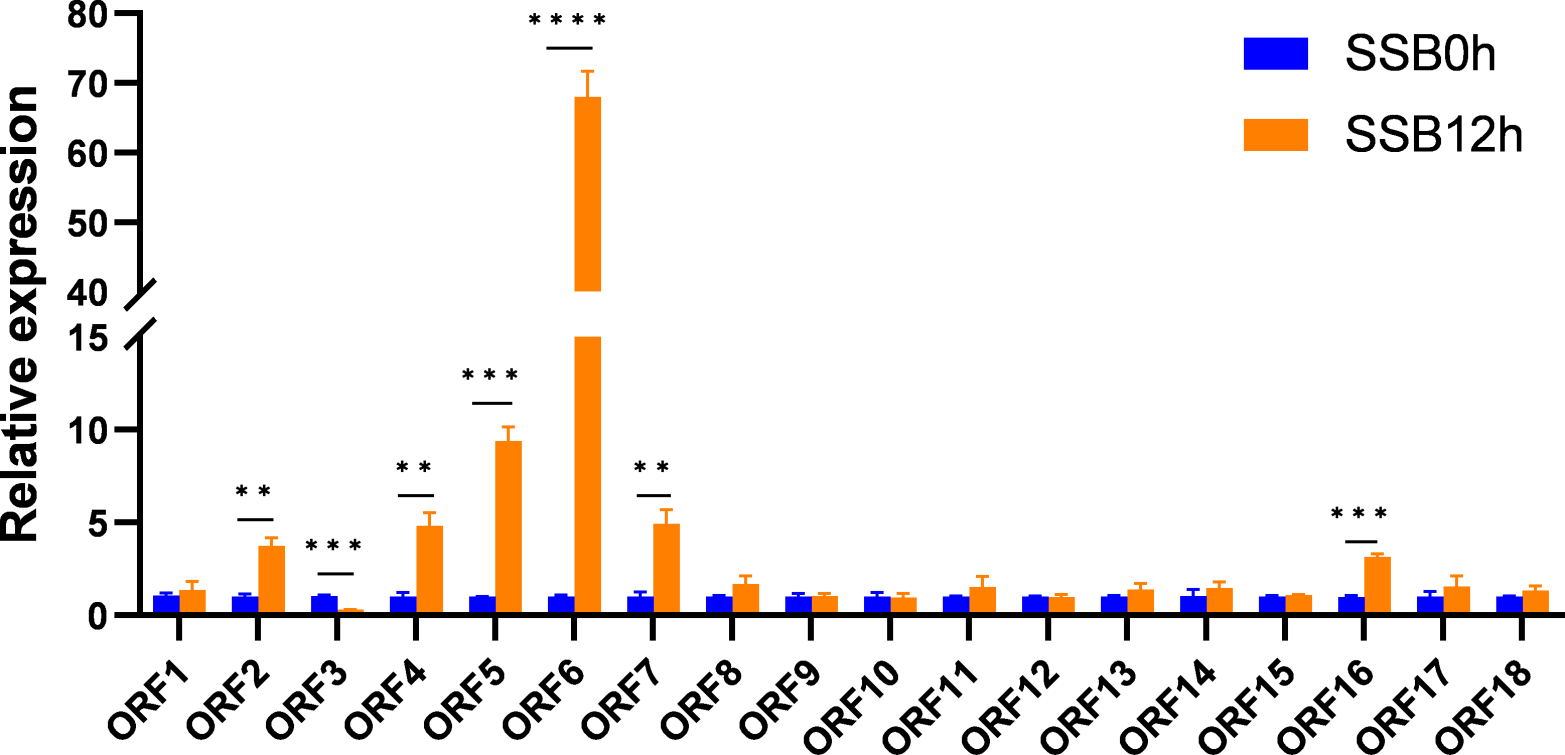
Expression levels of candidate genes at the *qRSSB4* locus in Aituogu151 during SSB invasion. (SSB0h): represents 0h before inoculation with SSB; (SSB12h): represents 12 h after inoculation with SSB. By conducting an Independent Samples t-test on the two sets of data, we can determine whether there is a significant difference between the two groups and obtain the *P*-value. The error line represents the standard error of three biological replicates **P < 0.01; ***P < 0.001; ****P < 0.0001).

A region of approximately 400 kb containing 65 genes was selected for candidate gene analysis. We annotated 65 genes using the Rice Genome Annotation Project database (http://rice.uga.edu). According to the functional annotation of genes and previous reports, 18 non transposon genes (ORF1-18) were selected as candidate genes, with gene IDs of *LOC_Os04g33950*, *LOC_Os04g33990*, *LOC_Os04g34000*, *LOC_Os04g34030*, *LOC_Os04g34050*, *LOC_Os04g34140*, *LOC_Os04g34250*, *LOC_Os04g34270*, *LOC_Os04g34300*, *LOC_Os04g34330*, *LOC_Os04g34360*, *LOC_Os04g34370*, *LOC_Os04g34390*, *LOC_Os04g34410*, *LOC_Os04g34420*, *LOC_Os04g34490*, *LOC_Os04g34590*, *LOC_Os04g34600*. To determine which gene was a hypothetical candidate gene, we examined the expression levels of these genes in Aituogu151 (An insect-resistant rice variety) in response to SSB invasion. The results showed that seven genes responded to SSB invasion (**Figure 4**). Among them, *ORF6* (*LOC_Os04g34140*) was significantly induced under the invasion of SSB, with the strongest response, followed by *ORF2* (*LOC_Os04g33990*), *ORF4* (*LOC_Os04g34030*), *ORF5* (*LOC_Os04g34050*), *ORF7* (*LOC_Os04g34250*), and *ORF16* (*LOC_Os04g34490*); However, *ORF3* (*LOC_Os04g34000*) was significantly downregulated under the invasion of SSB (**Figure 4**). Therefore, *ORF6* was most likely a candidate gene for the *qRSSB4* locus, named *OsRSSB4*, for further functional validation.

### 3.5 *OsRSSB4* positively regulates rice resistance to the SSB

**Figure 5:**
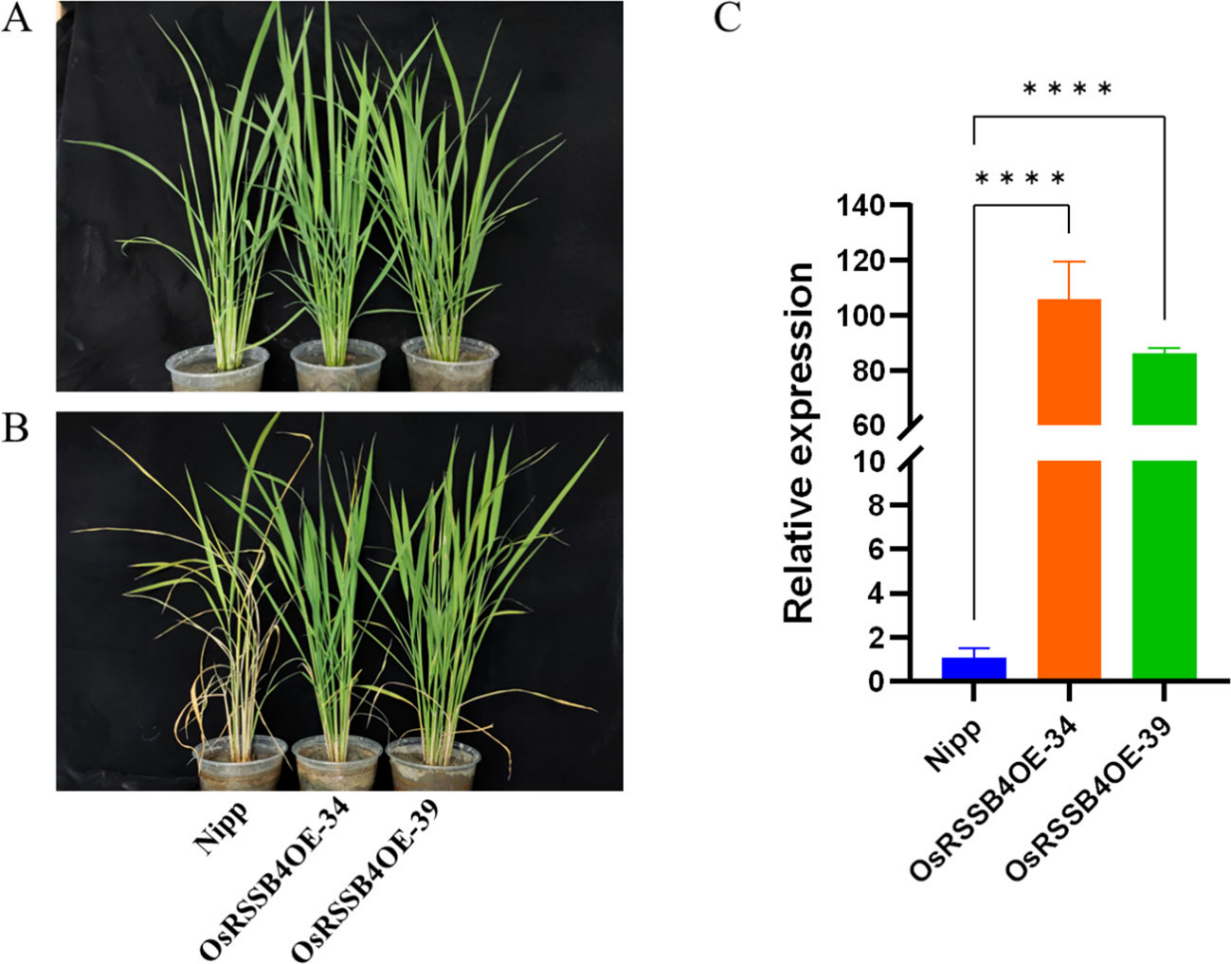
Identification of insect resistance of overexpressed variety (*OsRSSB4OE*). **(A)** Rice was inoculated with SSB for 0 days. **(B)** Rice plants were inoculated with SSB for 20 days. **(C)** Compositional expression levels of *OsRSSB4* in various rice plant lines. Perform a one-way ANOVA on the data from each group to determine if there are significant differences between them. If the one-way ANOVA indicates significant differences, use the pairwise comparison method of the Dunnett test to compare the differences between each treatment group and the control group (****P < 0.0001).

To further verify the involvement of *OsRSSB4* in defense regulation of rice against SSB, we adopted a transgenic approach. The expression of *OsRSSB4* was enhanced by expressing *OsRSSB4* under the control of the cauliflower mosaic virus (CaMV) 35S promoter (the recipient variety was Nipponbare). We obtained a stable *OsRSSB4* overexpressing variety (*OsRSSB4OE*) through four consecutive generations of screening. Compared with the wild type, the expression level of *OsRSSB4* in the *OsRSSB4OE* variety was 86–105 times higher (**Figure 5C**). Furthermore, we conducted bioassays on the insect resistance of the T4 generation *OsRSSB4OE* variety using live plants and detached stems, with the wild-type variety as a control. The results showed that, compared with the wild-type, the OsRSSB4OE variety exhibited good resistance to SSB (**Figures 5A, B**). After 20 days of inoculation with SSB, the withered heart rate of the wild type reached 100% (12/12), and the resistance level was 9. The withered heart rate of *OsRSSB4OE-34* and *OsRSSB4OE-39* varieties was only 8.3% (1/12) and 0% (0/14), with resistance levels of 1 and 0, respectively. Meanwhile, the detached stem feeding experiment also showed the same trend results; The results showed that compared with the wild type, the average corrected mortality rate of the SSB fed on the *OsRSSB4OE* variety reached 94.3%, and the resistance reached a high level (**Table 3**). Overall, it has been preliminarily confirmed that *OsRSSB4* is involved in the defense of rice against SSB, and positively regulates the resistance of rice to SSB.

**Table 3:** *In-vitro* bioassay of Nipponbare (S) and *OsRSSB4OE* lines (R) on SSB.

Nipponbare(S)			*OsRSSB4OE*(R)			
Treatment	Number of deaths	Mortality (%)	Treatment	Number of deaths	Corrected mortality	Resistance level
S1	2	20	R1	10	100%	0
S2	1	10	R2	10	100%	0
S3	0	0	R3	8	77.1%	1
S4	2	20	R4	10	100%	0
Average	–	12.5	Average	–	94.3%	0

## 4 Discussion

The ratio of the total number of withered seedlings and white heads to the number of tillers (damage index) is useful for evaluating the resistance of rice to SSB. It is well known that during the tillering stage of rice, when there are withered seedlings and white heads, it is generally believed that the rice plant is attacked by SSB (Xiang et al., 2023). Therefore, the number of withered seedlings and white heads may be used as indicators of rice resistance to SSB. The damage index was used as an indicator for the first time to evaluate the SSB resistance characteristics of 201 rice varieties at the tillering stage. Further confirmation of a significantly correlated locus (*qRSSB4*) with a damage index indicated that the number of withered seedlings and white heads is a useful data feature for evaluating rice resistance to SSB. Analogous to this quantitative characteristic may help in the genetic analysis of resistance to other pests in crops, such as rice. For example, the number of rolled leaves formed by a single leaf can be used as an indicator trait to evaluate the resistance of rice to the rice leaf roller; The number of row shaped pores in the new leaves of corn can be used as an indicator trait to evaluate the resistance of corn to corn borer. In addition, this pest trait is closely related to photosynthetic efficiency, transpiration, nutrient transport, and growth and development of plants, which greatly affects the yield and quality of crops. Therefore, in the future, combining pest traits with other high-throughput phenotypic techniques could serve as a method for evaluating the degree of pest impact on crops in the field.

*OsRSSB4* is an important member of the U-box protein family that regulates PTI and ETI signaling pathways in plant innate immune responses. The U-box protein in plants is much more abundant than in other organisms and is collectively referred to as the plant U-box protein (PUB). To date, 77 U-box proteins have been identified in rice, 64 U-box proteins have been identified in the model plant Arabidopsis, 101 U-box proteins have been identified in Chinese cabbage, and only 2 and 21 U-box proteins have been identified in yeast and humans, respectively (Hwang et al., 2015; Wang et al., 2015). The high PUB protein content in plants suggests that this type of protein plays multiple important roles in plant evolution. In *Arabidopsis*, under the stimulation of flagellin, the receptor kinase BAK1 phosphorylates PUB12 and PUB13, thereby activating the ubiquitination of flagellin receptor FLS2 by PUB12 and PUB13 to promote the degradation of its ubiquitin-26S proteasome pathway. This signal transduction ultimately leads to the negative regulation of flagellin FLS2 induced plant PTI resistance by PUB12 and PUB13 (Lu et al., 2011a; Zhou et al., 2015); The PUB protein MAC3A and MAC3B with WD40 repeat structure positively regulate the self-activated innate immune response phenotype of the dominant mutant *snc1* of the R protein SNC1. The absence of these two homologous proteins leads to a decrease in the originally increased SA content in the *snc1* mutant, and the transcription level of disease-related genes also decreases accordingly (Monaghan et al., 2009). In tomatoes, the PUB protein ACRE276 exhibits E3 ubiquitin ligase activity, and after silencing its gene, it specifically loses Avr9-Cf9 mediated HR (Yang et al., 2006). In tobacco, the PUB protein ACRE276 can be induced by mechanical damage, and the Avr9 effector protein is secreted by *Cladosporium fulvum*. Silencing the ACRE276 gene through RNAi technology can lead to the loss of HR mediated by Avr9-Cf9 and Avr4-Cf4 in tobacco, and HR stimulated by the tobacco mosaic virus (TMV) gene *N* and its non-toxic factor *p50* was also significantly reduced (Yang et al., 2006). In rice, the rice PUB protein SPL11 activates SPIN6 through ubiquitination degradation of Rho type GTPase. On the other hand, SPIN6 can inactivate the active small G protein OsRac1 and catalyze its hydrolysis, thereby inhibiting OsRac1 mediated innate immune response (Liu et al., 2015); Therefore, SPL11 regulates the PTI resistance of rice to PAMPs such as flagellin and chitin, thereby endowing rice with resistance to rice blast and bacterial blight (Zeng et al., 2008; Liu et al., 2015). In our study, multiple pieces of evidence have suggested that the PUB protein OsRSSB4 positively regulate effect on SSB resistance in rice. Firstly, the GWAS results indicated that *OsRSSB4* is associated with SSB resistance. Secondly, overexpression of *OsRSSB4* in transgenic rice significantly increased its resistance to SSB. Furthermore, according to the annotation, OsRSSB4 belongs to the presumed U-box protein CMPG1. The PUB protein CMPG1 in tobacco and tomatoes has E3 ubiquitin ligase activity. After silencing the *CMPG1* gene, Avr9-Cf9 mediated HR was lost and plant resistance to *C. fulvum* was significantly reduced. Additionally, the effector factors AvrPto secreted by *Pseudomonas syringae* and HR mediated by the R protein Pto were largely lost when the *CMPG1* gene was silenced (González-Lamothe et al., 2006). In recent years, research has found that the effector factor Avr3a of *Phytophthora infestans* inhibits ETI and PTI regulated by the CMPG1 protein by binding to it (Gilroy et al., 2011; Yaeno et al., 2011; Chaparro-Garcia et al., 2015). In summary, our data and previous research findings suggest that *OsRSSB4* may regulate the defense level of rice against SSB by regulating ETI and PTI.

This study found that in addition to *OsRSSB4* (*LOC_Os04g34140*), there are six other genes in the *qRSSB4* locus that respond to SSB invasion (**Figure 4**). Among them, *LOC_Os04g33990* encodes a harpin-induced protein 1 domain-containing protein, and reports indicate that *HIN1* is a gene related to resistance to anthracnose in sorghum (Upadhyaya et al., 2013). *LOC_Os04g34030* encodes E3 ubiquitin ligase, which has been cloned (gene symbol: *OsPUB34*). Research has shown that *OsPUB34* may positively regulate resistance to rice blast by accumulating reactive oxygen species (ROS) and increasing the expression levels of defense-related genes (Zhang et al., 2022). *LOC_Os04g34050* encodes a valine glucose (VQ) motif-containing protein. Hao et al. (2022). found that the OsVQ25 protein balances broad-spectrum disease resistance and plant growth by interacting with the U-Box E3 ligase OsPUB73 and the transcription factor *OsWRKY53*. *LOC_Os04g34250* encodes for serine/threonine-protein kinase receptor precursor. Serine/threonine protein kinases use ATP as a phosphate donor to catalyze the phosphorylation of serine or threonine residues on target proteins, including cyclin-dependent kinases, mitogen-activated protein kinases (MAPKs), protein kinase D, and DNA-dependent protein kinases. Evidence suggests that MAPK actively participates in the defense response of rice against SSB (Wang et al., 2013; Hu et al., 2015, 2018). *LOC_Os04g34490* encodes nodulin, which is one of the plant polypeptide complexes and has certain benefits for plants. In addition, *LOC_Os04g34000*, encoding digalactosyldiacylglycerol synthase (chloroplast precursor), was significantly downregulated under SSB intrusion. Compared with the wild-type, the content of JA, JA-Ile, and OPDA, as well as the expression level of JA response genes, were increased in the functionally deficient mutant of digalactosyldiacylglycerol synthase in *Arabidopsis*, and the phloem cells were lignified under normal growth conditions (Lin et al., 2016). These results confirm that *LOC_Os04g34000* may be involved in the regulation of SSB attacks in rice. In summary, our data provides important reference information for future functional research on SSB resistance genes in rice.

## Data Availability

Sequencing data of rice were deposited at the National Center for Biotechnology (NCBI) Sequence Read Archive (SRA) under bioproject PRJNA598020 (for genome re-sequencing).
